# Features and Prognostic Value of Quantitative Electroencephalogram Changes in Critically Ill and Non-critically Ill Anti-NMDAR Encephalitis Patients: A Pilot Study

**DOI:** 10.3389/fneur.2018.00833

**Published:** 2018-10-05

**Authors:** Nan Jiang, Hongzhi Guan, Qiang Lu, Haitao Ren, Bin Peng

**Affiliations:** Department of Neurology, Peking Union Medical College Hospital, Chinese Academy of Medical Sciences, Beijing, China

**Keywords:** anti-NMDAR encephalitis, quantitative EEG, amplitude-integrated EEG, prognosis, intensive care unit

## Abstract

Anti-N-methyl-D-aspartate receptor (NMDAR) encephalitis is a common cause of encephalitis in intensive care units. Until now, no reliable method has existed for predicting the outcome of anti-NMDAR encephalitis. In this study, we used quantitative electroencephalography (qEEG) to examine the brain function of anti-NMDAR encephalitis patients and assessed its predictive value. Twenty-six patients diagnosed with anti-NMDAR encephalitis were included and grouped according to whether they were treated in intensive care units (14 critically ill vs. 12 non-critically ill). All patients underwent 2-h 10-channel qEEG recordings at the acute stage. Parameters, including amplitude-integrated electroencephalogram (aEEG), spectral edge frequency 95%, total power, power within different frequency bands (δ, θ, α, and β), and percentages of power in specific frequency bands from frontal and parietal areas were calculated with NicoletOne Software and compared between groups. The short-term outcome was death or moderate/severe disability at 3 months after onset, measured with a modified Rankin Scale, and the long-term outcome was death, disability or relapse at 12 months. No differences in qEEG parameters were observed between the critically ill and non-critically ill patients. However, differential anterior-to-posterior alterations in δ and β absolute band power were observed. Logistic regression analysis revealed that a narrower parietal aEEG bandwidth was associated with favorable long-term outcomes (odds ratio, 37.9; *P* = 0.044), with an optimal cutoff value of 1.7 μV and corresponding sensitivity and specificity of 90.00 and 56.25%, respectively. In a receiver operating characteristic analysis, the area under the curve was 0.7312. In conclusion, the qEEG parameters failed to reflect the clinical severity of anti-NMDAR encephalitis. However, the parietal aEEG bandwidth may separate patients with favorable and poor long-term outcomes in early stages. The underlying mechanisms require further investigation.

## Introduction

Anti-N-methyl-D-aspartate receptor encephalitis is an autoimmune encephalitis involving antibodies directed against the NR1 subunit of the NMDA receptor (NMDAR), and is often associated with ovarian teratomas (1, 2). A considerable proportion of anti-NMDAR encephalitis patients fall into coma or develop status epilepticus and require intensive care. In one retrospective study, anti-NMDAR encephalitis accounted for 1% of all admissions of young adults to intensive care units (ICUs) (3).

Early identification of neurological outcomes is important in terms of therapeutic options. Several prognostic measures have been evaluated in anti-NMDAR encephalitis, including the Glasgow Coma Scale (GCS) score, number of complications, catatonia-predominant type, and electroencephalogram (EEG) (4–6). EEG is a commonly used monitoring tool at the bedside in the ICU, and an EEG “extreme delta brush” (EDB) pattern has been described in anti-NMDAR encephalitis and shown to be a marker of more severe disease and perhaps worse outcome (6). However, the identification of EDB on raw EEG requires experienced raters, which is inconvenient for the continuous monitoring of critically ill patients in the ICU. In addition, the prevalence of EDB is <30% in anti-NMDAR encephalitis patients; therefore, it may not be a sensitive prognostic factor (6–9). Hence, exploration for additional EEG markers to measure severity and predict the outcomes of anti-NMDAR encephalitis is needed, especially for cases where EDB is absent.

A variety of quantitative EEG (qEEG) parameters have been developed in neurocritical care practice, including some applied to the diagnosis of viral encephalitis (10). By applying fast Fourier transformation or other techniques, EEG can be quantified in terms of amplitude, power, frequency, and rhythmicity to generate numerical values, ratios, or percentages (11). Some qEEG parameters may provide quantitative information about short-term and long-term outcomes (11). In this study, we sought to explore features of brain background activity using quantitative analyses of EEG in anti-NMDAR encephalitis patients. The relationships of qEEG characteristics with disease outcomes were also assessed.

## Methods

### Participant enrollment, data collection, and follow-up

This single-center retrospective observational study was approved by the ethics committee of Peking Union Medical College Hospital. Eligible patients were enrolled from April 2014 to May 2017. Patient consent was not required because de-identified data were used in this study.

All enrolled patients met the diagnostic criteria for anti-NMDAR encephalitis introduced in 2016 (12). Exclusion criteria were as follows: (1) age at onset <12 years; (2) identifiable intracranial infections, other autoimmune encephalitis, or other etiologies causing admission to the ICU; (3) inability to cooperate with qEEG monitoring, due to issues such as agitation; (4) known medical history of a severe neurological deficit [modified Rankin Scale (mRS), ≥2] before onset of anti-NMDAR encephalitis; (5) lack of qEEG recording during the first week of hospitalization in our institute; and (6) missing data or loss to follow-up. According to the severity of disease, patients were further divided into critically ill or non-critically ill subgroups. Patients enrolled in the critically ill subgroup satisfied the following criteria: treated in the ICU for at least 48 h because of (1) GCS ≤ 8, (2) status epilepticus, or (3) hypoventilation or severe autonomic dysfunction. Patients who did not meet these criteria comprised the non-critically ill subgroup.

We collected the demographic, clinical and laboratory data of patients, including symptoms at the acute stage, serum and CSF studies, and therapeutic regimens. The short-term outcome for this study was death or degrees of disability, which was evaluated with the mRS at 3 months after onset. Patients were considered to have a favorable short-term outcome when their mRS scores were ≤ 2 without increasing compared with baseline, and poor short-term outcome if the mRS scores were ≥3 or had increased. Long-term outcomes were obtained from hospital medical records or face-to-face interviews 12 months after onset. Patients with mRS scores ≥3 for the whole experimental period or experienced relapse events and received another episode of first-line immunotherapy were defined as experiencing poor long-term outcomes. Patients were considered to have favorable long-term outcomes if their mRS scores were ≤ 2 with no relapse events.

In the final analysis, a total of 26 patients completed 1-year follow-up. Of these, 25 patients were diagnosed with definite anti-NMDAR encephalitis, and 1 patient met the diagnostic criteria for antibody-negative anti-NMDAR encephalitis. We also recruited 10 healthy volunteers with similar ages and collected their qEEG information as a control group.

### EEG recording and interpretation

qEEG monitoring was performed for at least 2 h for each patient during the first week after admission to our center. Medication administrations during EEG recording were noted and intravenous anti-epileptic agents as well as sedatives were suspended before the start of qEEG monitoring. Silver-chloride disc electrodes were placed according to the International 10–20 System, with a 10-channel layout at the Fp1, F3, C3, P3, O1, and Fp2, F4, C4, P4, O2 sites. The reference electrode was located at Cz, and Fz was used as ground. A 1.0-Hz low- and 35-Hz high-frequency filter was used. Impedances were maintained below 10 kΩ. The qEEG recording was performed using a NicoletOne EEG monitor (VIASYS Healthcare Inc.), and both raw EEG and processed qEEG tracings were sampled simultaneously. The aEEG, spectral edge frequency 95% (SEF-95), total power, power within δ, θ, α, and β frequency bands, as well as relative percentages of power in specific frequency bands were calculated automatically and exported via NicoletOne system.

All qEEG recordings were analyzed off-line. For each patient, we selected one 30-min artifact-free epoch manually from the F3-F4 and P3-P4 montage for further quantitative analysis. We selected F3-F4 as the representative area of the anterior cross-cerebral EEG signal, and P3-P4 as the representative area of the posterior cross-cerebral EEG signal. Because signals from Fp1-Fp2 and O1-O2 often contain more artifacts or higher impedances, we did not select these recordings for final quantitative analysis.

### Statistical analysis

Descriptive data are presented as mean ± standard deviation (SD) or median with interquartile ranges (IQR). We use Student's *t*-test or the Mann-Whitney *U*-test for group comparisons of continuous variables, and Fisher's exact test for categorical variables, as appropriate. The Kruskal-Wallis test with Bonferroni correction was used for multiple comparisons. Wilcoxon signed rank tests were performed to compare brain activity between anterior and posterior regions. Univariate and multivariate logistic regression with stepwise estimation method was performed to find the independent predictive ability of outcome predictors. Receiver operator characteristic (ROC) curves and areas under the curve (AUC) were constructed to study the ability of aEEG to predict outcomes. For these 2-tailed tests, *p* < 0.05 was considered statistically significant. Statistical analyses were performed using Stata software version 14.1.

## Results

### Patient characteristics

Of the 26 patients, 11 (42.3%) were male and 15 (57.7%) were female. The median age was 20 (IQR: 16–27) years. No tumor was found in any of the male patients. Eight (53%) female patients had underlying tumors, which were pathologically confirmed as ovarian teratomas. All patients received intravenous immunoglobulin (2 g/kg divided for 5 days), 25 (96.2%) treated with methylprednisolone (1 g/d for at least 3 days), and 14 (53.8%) patients received second-line immunotherapy. Table 1 summarizes the comparison of demographic and clinical information between critically ill and non-critically ill patients.

**Table 1:** Comparison of demographic, clinical, and CSF characteristics between critically ill and non-critically ill patients.

	**Critically Ill subgroup**	**Non-critically Ill subgroup**	***P*-value**
**DEMOGRAPHIC**			
Gender(female)	8/14	7/12	1
Age	20(15,26)	20.5(17,31)	0.502
**CLINICAL INFORMATION**			
Fever	13/14	4/12	0.003^*^
Headache	10/14	4/12	0.113
Psychiatric behaviour	12/14	11/12	1
Cognition dysfunction	4/14	6/12	0.422
Memory impairment	4/14	9/12	0.047^*^
Speech dysfunction	4/14	8/12	0.113
Seizures	13/14	11/12	1
Movement disorder	9/14	6/12	0.692
Central hypoventilation	7/14	0/12	0.006^*^
Autonomic dysfunction	10/14	5/12	0.233
Decreased consciousness	11/14	3/12	0.016^*^
Glasgow Coma Scale	5(3,6)	11.5(7,15)	0.005^*^
Days till diagnosis	20(15,25)	17.5(13.5,34.5)	0.959
Days in hospital	61(54,120)	16.5(13,27.5)	0.0002^*^
Mechanical ventilation	10/14	0/12	0.0001^*^
Tumor	7/14	1/12	0.036^*^
Tumor in female	7/8	1/7	0.01^*^
Elevated CSF protein	2/14	5/12	0.19
CSF leukocyte			0.728
~5	8/14	6/12	
6~50	5/14	5/12	
51~	1/14	1/12	
Oligoclonal band			0.642
Negative	6/13	3/10	
Suspected	0/13	5/10	
Positive	7/13	2/10	
Antibody titers in CSF			0.324
~1:10	1/14	1/12	
1:32	3/14	5/12	
1:100~	10/14	6/12	
Antibody titers in serum			0.040^*^
Negative	3/14	7/12	
1:10	2/14	1/12	
1:32~	9/14	4/12	
**SECOND-LINE IMMUNOTHERAPY**			
MMF	9/14	4/12	0.238
MTX	4/14	0/12	0.100
CTX	1/14	0/12	1
RTX	1/14	1/12	1
No 2nd-line Immunotherapy	4/14	8/12	0.113

* *p < 0.05*.

*CSF, cerebrospinal fluid; MMF, mycophenolate mofetil; MTX, methotrexate; CTX, cyclophosphamide; RTX, rituximab*.

### Patient outcomes

Patient short-term outcomes were as follows: favorable outcome, 7 of 26 (26.9%); and poor outcome, 19 of 26 (73.1%). There was no significant difference between the two subgroups in terms of demographic information, CSF profiles, concomitant tumors, or immunotherapy regimens. All patients in the favorable short-term outcome subgroup had symptoms of impaired memory at admission (7/7 vs. 6/19, *p* = 0.005), and patients with impaired consciousness on admission were more likely to have poor short-term outcomes (1/7 vs. 13/19, *p* = 0.026). Short-term outcomes of the critically ill subgroup were worse than those of the non-critical subgroup (1/7 vs. 13/19, *p* = 0.026).

There were 10 (38.5%) patients experiencing poor long-term outcomes, including 5 with mRS ≥3 and 7 with relapse events within 12 months. One patient died because of complications due to infection. There were no significant differences between the favorable long-term outcome group and poor long-term outcome group in terms of sex, age, clinical symptoms, severity of onset, duration of hospital stay, profiles in CSF, concomitant tumors, or immunotherapy regimens during hospitalization.

### qEEG findings

The detailed results for qEEG parameters are presented in Table 2. Compared with the healthy control group, the δ relative band power in the posterior area was significantly increased (*p* = 0.024) in the anti-NMDAR patient group, while β relative (*p* = 0.006) and β absolute (*p* = 0.008) band power in the posterior area were significantly reduced. The qEEG parameters in anterior area showed no significant differences between the patient group and the control group. There were also no significant differences in qEEG parameters between critically ill and non-critically ill subgroups, or between patients with favorable and poor short-term outcomes. However, in terms of long-term outcomes, the aEEG bandwidth in the parietal area was significantly lower in patients with favorable outcomes than those with poor outcomes (1.6 vs. 1.95 μV, *p* = 0.030). The parietal total power was also significantly lower in the favorable long-term outcome subgroup compared with the poor outcome subgroup (10.68 vs. 27.9 μV^2^, *p* = 0.022).

**Table 2:** Comparisons of qEEG parameters between patients and controls, critically ill patients and non-critically ill patients as well as patients with different outcomes.

		**Patients group**	**Control group**	***P*-value**	**Critically Ill subgroup**	**Non-critically Ill subgroup**	***P*-value**	**Favorable short-term outcome**	**Poor short-term outcome**	***P*-value**	**Favorable long-term outcome**	**Poor long-term outcome**	***P*-value**
Frontal area	aEEG Upper Margin (μV)	10.9(9.1,12.4)	10.8(10,11.4)	0.860	11.4(8.2,12.8)	10.8(9.7,11.9)	0.918	11(9.1,12.1)	10.7(8.8,12.8)	0.563	10.2(8.3,12.25)	11(10,12.5)	0.257
	aEEG Lower Margin(μV)	8.95(7.4,10.3)	8.9(7.9,9)	0.671	9.3(6.7,11)	8.8(7.9,9.8)	0.959	9(7.4,9.7)	8.9(7.2,11)	0.544	8.4(6.7,10.1)	9.2(8.2,10.3)	0.304
	aEEG Bandwidth(μV)	1.7(1.6,2)	1.9(1.8,2.1)	0.069	1.65(1.5,2)	1.7(1.65,1.95)	0.405	1.8(1.6,2)	1.7(1.6,2)	0.539	1.65(1.55,1.85)	1.8(1.7,2)	0.150
	SEF-95(Hz)	1.31(1.24,1.42)	1.39(1.35,1.4)	0.304	1.32(1.29,1.44)	1.31(1.21,1.41)	0.552	1.4(1.31,1.44)	1.31(1.17,1.4)	0.117	1.32(1.3,1.43)	1.3(1.15,1.4)	0.290
	Total Power(μV)	27.86(19.25,61.56)	18.92(16.88,26.01)	0.120	27.04(11.21,64.15)	29.58(20.85,48.95)	0.662	27.86(16.76,35.09)	27.86(19.41,64.59)	0.272	24.82(15.23,50.87)	31.475(25,66.96)	0.257
	δ RBP(%)	56.45(40.9,65.39)	54.54(41.08,55.98)	0.397	49.22(39.46,58.37)	61.46(52.31,69.94)	0.143	54.75(39.46,57.53)	58.05(40.9,68.09)	0.563	55.235(40.18,66.43)	57.615(49.86,65.39)	0.732
	θ RBP(%)	15.11(11.76,21.13)	16.21(15.32,20.44)	0.572	19.27(11.6,23.9)	14.28(12.54,19.18)	0.700	14.25(11.6,20.61)	17.74(11.76,23.9)	0.603	17.42(12.04,22.515)	14.28(11.21,20.61)	0.544
	α RBP(%)	6.18(4.31,10.08)	8.59(7.4,11.56)	0.072	6.59(5.25,11.16)	6.18(4.24,8.56)	0.625	7.86(6.11,10.08)	5.98(4.08,10.42)	0.203	6.03(4.195,8.84)	7.61(5.98,11.16)	0.236
	β RBP(%)	10.68(7.44,22.25)	16.38(11.55,18.34)	0.340	12.07(7.54,22.57)	10.60(6.52,16.15)	0.440	14.8(12.01,22.28)	8.34(7.34,22.25)	0.272	10.93(7.49,23.705)	10.175(5.43,20.77)	0.399
	δ ABP(μV)	15.01(8.71,24.34)	8.36(7.03,14.01)	0.148	10.83(6.45,37.88)	16.54(9.88,20.75)	0.520	9.39(8.71,21.6)	16.5(8.67,37.88)	0.418	12.92(7.9,22.97)	16.59(8.71,37.88)	0.510
	θ ABP(μV)	4.125(2.5,6.93)	3.575(2.39,4.36)	0.427	4.48(1.87,10.84)	4.13(2.58,6.17)	0.939	3.05(2.48,5.57)	4.23(2.5,10.84)	0.355	3.28(2.18,8.16)	4.30(3.06,5.57)	0.580
	α ABP(μV)	1.915(0.79,4.82)	2.03(1.34,2.64)	0.659	2.17(0.56,5.26)	1.92(0.89,2.51)	0.857	2.26(0.84,2.68)	1.08(0.78,5.26)	0.885	0.89(0.59,4.35)	2.43(1.08,4.82)	0.197
	β ABP(μV)	3.1(1.64,4.25)	3.01(2.01,4.27)	0.646	3.43(1.55,5.18)	2.14(1.70,4.11)	0.738	3.08(1.98,4.25)	3.12(1.55,5.18)	0.862	2.58(1.60,4.18)	3.37(1.98,5.18)	0.693
Parietal area	aEEG Upper Margin (μV)	10.2(8.8,12.4)	11.4(10.2,13.6)	0.223	9.1(8,12.1)	10.5(9.6,12.6)	0.537	10.8(8.9,12.8)	10.1(8,12.1)	0.506	9.35(7.8,12.4)	10.95(10.3,12.4)	0.108
	aEEG Lower Margin(μV)	8.45(6.8,10.2)	9.45(8.2,11.4)	0.244	7.25(6.5,10.2)	8.6(7.65,10.35)	0.589	9.1(7.2,10.7)	8.3(6.5,10.2)	0.623	7.45(6.35,10.45)	9.2(8.6,10)	0.170
	aEEG Bandwidth(μV)	1.7(1.5,2)	1.9(1.8,2.1)	0.130	1.65(1.5,2.1)	1.8(1.66,2)	0.393	1.9(1.6,2)	1.7(1.5,2)	0.398	1.6(1.5,1.8)	1.95(1.7,2.1)	0.030
	SEF-95(Hz)	1.29(1.19,1.4)	1.38(1.35,1.4)	0.082	1.36(1.24,1.4)	1.23(1.19,1.34)	0.104	1.33(1.22,1.4)	1.29(1.19,1.4)	0.622	1.36(1.21,1.43)	1.24(1.18,1.29)	0.057
	Total Power(μV)	18.88(10.58,33.86)	23.57(14.63,35.69)	0.698	16.51(10.58,43.39)	22.68(10.38,27.9)	0.857	13.84(10.13,28.43)	24.2(10.63,43.39)	0.470	10.68(9.33,26.47)	27.9(21.16,43.39)	0.022
	δ RBP(%)	49.75(41.61,65.05)	39.715(29.78,48.56)	0.024^*^	51.05(41.61,66.5)	49.75(38.29,61.95)	0.625	49.47(33.66,51.63)	53.04(42.91,67.91)	0.184	49.75(42.66,61.95)	57.11(39.87,67.91)	0.732
	θ RBP(%)	16.545(12.94,21.77)	15.72(14.95,17.62)	0.724	16.55(12.94,21.77)	16.69(13.31,21.06)	0.857	15.84(12.07,21.93)	17.04(13.44,21.77)	0.686	16.55(13.19,22.19)	16.69(12.07,20.18)	0.772
	α RBP(%)	7.995(5.26,14.93)	13.865(10.14,14.9)	0.066	7.09(4.91,11.94)	12.68(6.78,21.14)	0.143	13.02(10.5,27.48)	7.53(4.43,14.37)	0.073	7.66(4.84,14.65)	9.64(6.65,20.46)	0.414
	β RBP(%)	9.765(6.82,15.27)	17.44(14.79,26.7)	0.006^*^	11.9(8.79,16.62)	8.575(5.865,12.57)	0.165	10.83(6.82,14.89)	9.47(6.11,15.65)	0.644	10.68(8.09,18.48)	8.17(6.11,13.87)	0.114
	δ ABP(μV)	10.095(5.6,13.72)	7.015(3.63,13.56)	0.458	11.5(5.7,20.86)	8.46(5.39,12.73)	0.520	6.61(4.74,11.73)	11.26(5.7,17.38)	0.272	6.48(4.82,12.18)	11.76(9.73,20.86)	0.054
	θ ABP(μV)	2.89(1.47,6.32)	3.675(2.36,5.61)	0.778	2.39(1.35,6.45)	3.48(1.74,6.14)	0.857	2.83(1.35,7.68)	2.95(1.58,6.32)	0.908	1.95(1.34,6.39)	3.83(2.83,6.01)	0.257
	α ABP(μV)	1.755(0.67,4.95)	3.17(1.56,8.07)	0.148	1.16(0.55,4.38)	2.19(1.2,5.63)	0.396	2.38(1.4,6.31)	1.45(0.55,4.38)	0.355	1.17(0.5,4.67)	2.61(1.99,6.31)	0.133
	β ABP(μV)	1.75(1.04,3.05)	4.23(2.63,5.97)	0.008^*^	2.15(1.04,3.05)	1.33(1.04,2.77)	0.410	2.48(1.43,3.05)	1.58(1.03,3.05)	0.402	1.56(1.01,2.82)	2.24(1.22,3.1)	0.292

* *p < 0.05*.

*aEEG, amplitude-integrated electroencephalogram; RBP, relative band power; ABP, absolute band power; SEF-95, spectral edge frequency 95%*.

We also investigated whether the differences in qEEG parameters between anterior and posterior areas were correlated with severities or outcomes (see Table 3). In the healthy control group, we found that the aEEG lower margin, δ relative band power, α relative band power, and α absolute band power differed statistically between the anterior and posterior areas. The aEEG lower margin, α relative band power, and α absolute power exhibited a decline from anterior to posterior areas, while δ relative band power showed the opposite trend. However, in the patient group, the differences in aEEG lower margin, δ relative band power, and α absolute power vanished. The gradient of α relative band power still remained, and new gradients of δ absolute band power and β absolute band power appeared in the patient group. The gradients of α relative band power, δ absolute band power and β absolute band power existed in the non-critically ill subgroup, whereas the differences in all parameters disappeared in critically ill subgroup.

**Table 3:** The anterior-to-posterior gradient of qEEG parameters in patients and control group.

	**Patients group**			**Critically Ill subgroup**			**Non-critically Ill subgroup**			**Control group**			**Poor Long-term outcome**			**Favorable long-term outcome**		
	**Anterior area**	**Posterior area**	***P*-value**	**Anterior area**	**Posterior area**	***P*-value**	**Anterior area**	**Posterior area**	***P*-value**	**anterior area**	**Posterior area**	***P*-value**	**Anterior area**	**Posterior area**	***P*-value**	**Anterior area**	**Posterior area**	***P*-value**
aEEG upper margin	10.85 (9.1,12.4)	10.2 (8.8,12.4)	0.341	11.4 (8.2,12.8)	9.1 (8,12.1)	0.245	10.8 (9.7,11.85)	10.5 (9.6,12.6)	1.000	10.8 (10,11.4)	11.4 (10.2,13.6)	0.103	11 (10,12.5)	10.95 (10.3,12.4)	0.959	10.2 (8.3,12.25)	9.35 (7.8,12.4)	0.224
aEEG lower margin	8.95 (7.4,10.3)	8.45 (6.8,10.2)	0.162	9.3 (6.7,11)	7.25 (6.5,10.2)	0.124	8.8 (7.9,9.8)	8.6 (7.65,10.35)	0.753	8.9 (7.9,9)	9.45 (8.2,11.4)	0.047	9.2 (8.2,10.3)	9.2 (8.6,10)	0.759	8.4 (6.7,10.1)	7.45 (6.35,10.45)	0.127
aEEG Bandwidth	1.7 (1.6,2)	1.7 (1.5,1.5)	0.918	1.65 (1.5,2)	1.65 (1.5,2.1)	0.777	1.7 (1.65,1.95)	1.8 (1.65,2)	0.811	1.9 (1.8,2.1)	1.9 (1.8,2.1)	1.000	1.8 (1.7,2)	1.95 (1.7,2.1)	0.603	1.65 (1.55,1.85)	1.6 (1.5,1.8)	0.498
SEF-95	1.31 (1.24,1.42)	1.29 (1.19,1.4)	0.258	1.32 (1.29,1.44)	1.36 (1.24,1.4)	1.000	1.31 (1.205,1.41)	1.23 (1.185,1.34)	0.091	1.39 (1.35,1.4)	1.38 (1.35,1.4)	0.959	1.3 (1.15,1.4)	1.24 (1.18,1.29)	0.240	1.32 (1.3,1.43)	1.36 (1.21,1.43)	0.569
Total power	27.86 (19.25,61.56)	18.88 (10.58,33.86)	0.059	27.04 (11.21,64.15)	16.51 (10.58,43.39)	0.433	29.58 (20.845,48.95)	22.68 (10.38,27.9)	0.050	18.915 (16.88,26.01)	23.57 (14.63,35.69)	0.959	31.475 (25,66.96)	27.9 (21.16,43.39)	0.285	24.82 (15.23,50.87)	10.68 (9.33,26.47)	0.070
δ RBP	56.45 (40.9,65.39)	49.75 (41.61,65.05)	0.638	49.22 (39.46,58.37)	51.045 (41.61,66.5)	0.158	61.46 (52.305,69.94)	49.75 (38.285,61.945)	0.060	54.535 (41.08,55.98)	39.715 (29.78,48.56)	0.005	57.615 (49.86,65.39)	57.11 (39.87,67.91)	0.721	55.235 (40.18,66.43)	49.75 (42.66,61.95)	0.756
θ RBP	15.105 (11.76,21.13)	16.545 (12.94,21.77)	0.409	19.265 (11.6,23.9)	16.545 (12.94,21.77)	0.778	14.28 (12.54,19.175)	16.685 (13.305,21.055)	0.136	16.21 (15.32,20.44)	15.72 (14.95,17.62)	0.333	14.28 (11.21,20.61)	16.69 (12.07,20.18)	0.799	17.42 (12.04,22.515)	16.55 (13.19,22.19)	0.255
α RBP	6.18 (4.31,10.08)	7.995 (5.26,14.93)	0.002	6.59 (5.25,11.16)	7.09 (4.91,11.94)	0.363	6.18 (4.235,8.56)	12.675 (6.78,21.14)	0.002	8.585 (7.4,11.56)	13.865 (10.14,14.9)	0.005	7.61 (5.98,11.16)	9.64 (6.65,20.46)	0.114	6.03 (4.195,8.84)	7.66 (4.84,14.65)	0.005
β RBP	10.675 (7.44,22.25)	9.765 (6.82,15.27)	0.228	12.07 (7.54,22.57)	11.9 (8.79,16.62)	0.470	10.595 (6.52,16.15)	8.575 (5.865,12.57)	0.388	16.38 (11.55,18.34)	17.44 (14.79,26.7)	0.075	10.175 (5.43,20.77)	8.17 (6.11,13.87)	0.241	10.93 (7.49,23.705)	10.68 (8.09,18.48)	0.501
δ ABP	15.01 (8.71,24.34)	10.095 (5.6,13.72)	0.025	10.83 (6.45,37.88)	11.495 (5.7,20.86)	0.363	16.535 (9.875,20.745)	8.46 (5.385,12.725)	0.015	8.36 (7.03,14.01)	7.015 (3.63,13.56)	0.169	16.59 (8.71,37.88)	11.76 (9.73,20.86)	0.169	12.92 (7.9,22.97)	6.48 (4.82,12.18)	0.049
θ ABP	4.125 (2.5,6.93)	2.89 (1.47,6.32)	0.086	4.475 (1.87,10.84)	2.39 (1.35,6.45)	0.198	4.125 (2.58,6.17)	3.48 (1.735,6.135)	0.272	3.575 (2.39,4.36)	3.675 (2.36,5.61)	0.799	4.30 (3.06,5.57)	3.83 (2.83,6.01)	0.799	3.28 (2.18,8.16)	1.95 (1.34,6.39)	0.063
α ABP	1.915 (0.79,4.82)	1.755 (0.67,4.95)	0.304	2.17 (0.56,5.26)	1.155 (0.55,4.38)	0.778	1.915 (0.89,2.505)	2.185 (1.195,5.63)	0.071	2.03 (1.34,2.64)	3.17 (1.56,8.07)	0.047	2.43 (1.08,4.82)	2.61 (1.99,6.31)	0.445	0.89 (0.59,4.35)	1.17 (0.5,4.67)	0.535
β ABP	3.1 (1.64,4.25)	1.75 (1.04,3.05)	0.020	3.43 (1.55,5.18)	2.15 (1.04,3.05)	0.300	2.14 (1.695,4.105)	1.325 (1.04,2.765)	0.019	3.01 (2.01,4.27)	4.23 (2.63,5.97)	0.114	3.37 (1.98,5.18)	2.24 (1.22,3.1)	0.093	2.58 (1.60,4.18)	1.56 (1.01,2.82)	0.088

*aEEG, amplitude-integrated electroencephalogram; RBP, relative band power; ABP, absolute band power; SEF-95, spectral edge frequency 95%*.

**Figure 1:**
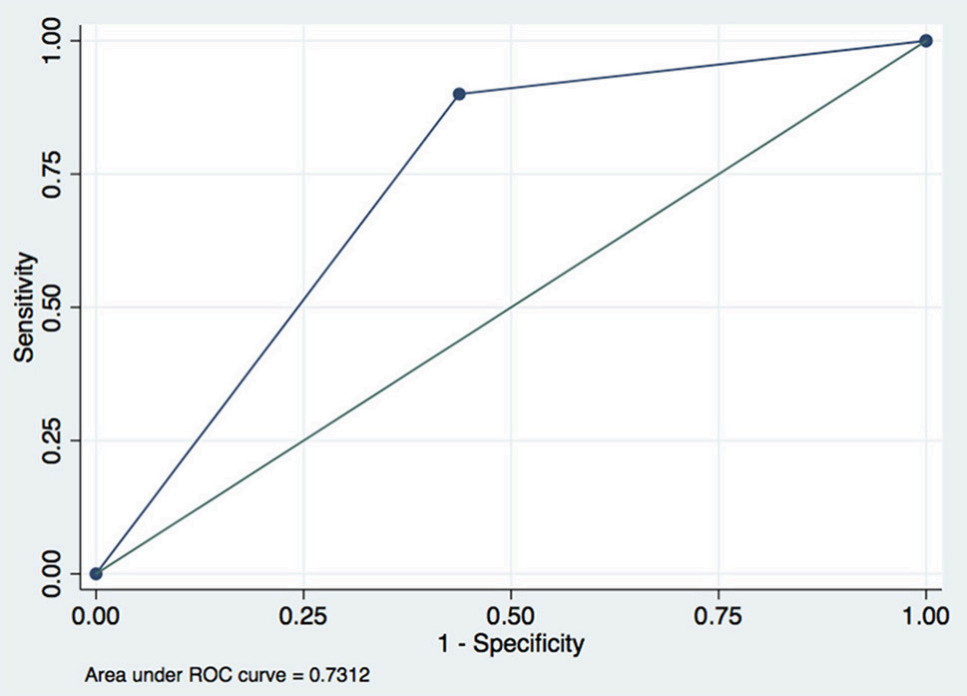
Receiver operating characteristic curve of parietal aEEG bandwidth predicting long-term outcomes of anti-NMDAR encephalitis when cutoff point is 1.7.

Taking the long-term outcome as dependent variable, with univariate logistic regression we screened parietal aEEG upper margin, aEEG bandwidth, SEF-95, and β relative band power as independent variables. Subsequent multivariate logistic regression analysis yielded only one predictor: the parietal aEEG bandwidth (odds ratio, 37.9; 95% confidence interval, 1.11–1295.27; *p* = 0.044). The maximal index of Youden was 1.4625 for a cutoff value of 1.7 μV, with a sensitivity and specificity of 90.00% and 56.25%, respectively. ROC analysis of parietal aEEG bandwidth yielded an area under the curve of 0.7312 (95% CI: 0.572–0.891; Figure 1). Furthermore, using another univariate logistic regression analysis, we found that parietal aEEG bandwidth was associated with long-term moderate/severe disability (mRS score ≥3) at 12 months (odds ratio, 761.88; 95% confidence interval, 1.53–378836.40; *p* = 0.036), but not associated with relapse events (*p* = 0.611).

## Discussion

Anti-NMDAR encephalitis has been recognized as a common cause of encephalitis in ICU. Despite its responsiveness to immunotherapy and tumor removal, the mortality rate of anti-NMDAR encephalitis in the ICU is 4–25% (4, 13–15). At present, there is no reliable tool for predicting outcomes of anti-NMDAR encephalitis. Most previous electrophysiological studies focused on raw EEG manifestations of anti-NMDAR encephalitis (6, 8, 9). In this study, we investigated the characteristics of qEEG in patients with anti-NMDAR encephalitis. To the best of our knowledge, this is the first study that depicts qEEG findings of patients with anti-NMDAR encephalitis. Our results indicate that most qEEG parameters, including aEEG background, total power, SEF-95, and power of various frequency bands of brain rhythms failed to measure the clinical severity of anti-NMDAR encephalitis. However, the widening of parietal aEEG bandwidth can be used as an objective marker to predict poor long-term outcomes with a good sensitivity.

**Figure 2:**
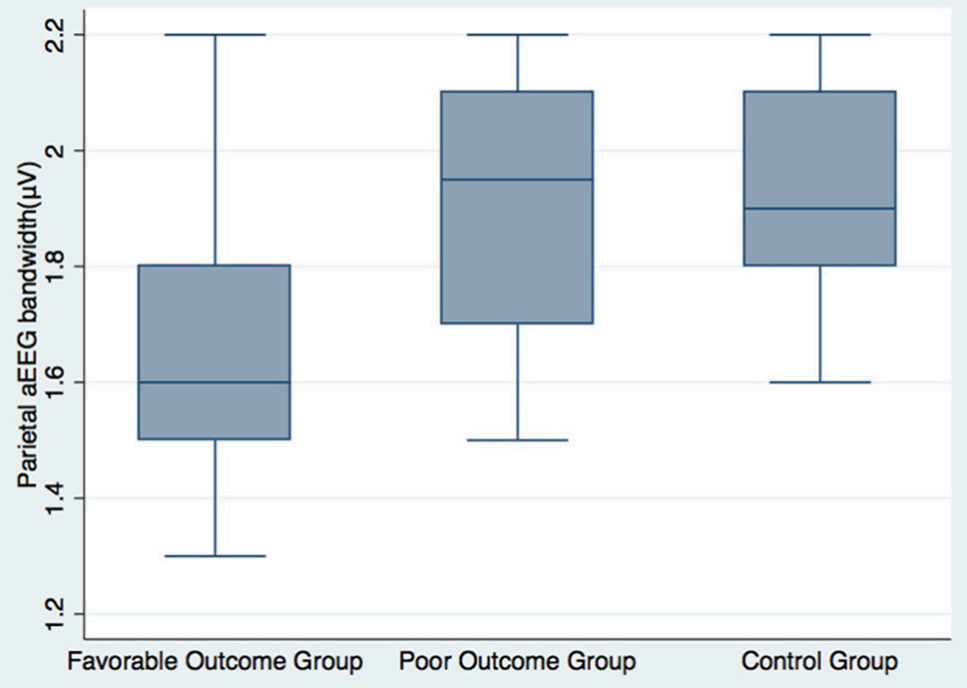
Parietal aEEG bandwidth among favorable long-term patients, poor long-term patients, and healthy volunteers.

aEEG is a type of processed EEG that is compressed with respect to amplitude and time, and the upper and lower margins of the aEEG reflect the maximum/minimum peak-to-peak amplitudes of the EEG signals (16). An abnormal aEEG, especially its lower margins, has been shown to be predictive of persistence of severe cerebral injury and poor neurologic outcome (17–19). However, aEEG bandwidth is not a commonly used monitoring marker. The prognostic value of parietal aEEG bandwidth in anti-NMDAR encephalitis is a novel finding and difficult to explain. Previous studies on aEEG have mainly concentrated on disorders that lead to neuron damage, such as stroke, traumatic brain injury, or hypoxic encephalopathy, which can be appraised by aEEG lower margins. However, anti-NMDAR encephalitis selectively reduces NMDAR function and changes the synaptic activities of neuronal networks without any impairment of other synaptic processes (20). Aberrant functioning of a single ion channel may result in various pathophysiological processes (20); therefore, the degree of NMDAR hypofunction may not have a linear relationship with EEG discontinuity. Thus, the subsequent aEEG changes may be difficult to evaluate using traditional aEEG lower margin. On the contrary, unlike simply measuring the lower margin, aEEG bandwidth may play a diagnostic and predictive role in disorders that are pathogenic to synapses. Specifically, the parietal aEEG bandwidth in healthy controls was wider than in patients with favorable long-term outcomes, and narrower than in those with poor long-term outcomes (Figure 2). This opposite trend suggests that the long-term outcome of anti-NMDAR encephalitis may be determined by underlying, unexplained pathophysiological mechanisms, which might be reflected in electrophysiological parietal aEEG bandwidth. The molecular mechanism underlying this requires further investigation.

Interestingly, compared with healthy controls, the lower margins of the aEEG in both the critically ill and non-critically ill subgroup did not show any significant differences. One possible reason for this phenomenon is the small number of patients. Nevertheless, it may also suggest that brain function in anti-NMDAR encephalitis is relatively intact even in critically ill patients. This characteristic of qEEG is potentially consistent with the pathogenesis of anti-NMDAR encephalitis. NMDAR is an ionotropic glutamate receptor distributed in entire brain tissues. Antibodies directed at the NR1 subunit of the NMDA receptors act by mechanisms including the binding, capping, and cross-linking of NMDA receptors, leading to internalization from the cell membrane surface and a selective decrease in NMDA receptor currents with no effect on synapse number or other synapse proteins (2, 21). The NMDAR hypofunction is non-destructive and reversible, which may explain why the lower margins of aEEG did not decline in the patient group.

Compared with the healthy control group, there were specific anterior-to-posterior graded alterations of qEEG parameters in patients with anti-NMDAR encephalitis. In particular, there were alterations in δ and β absolute band power. δ absolute band power in the posterior area was lower than in the anterior area in the healthy control group and non-critically ill subgroup, but higher than in anterior area of the critically ill subgroup. However, this trend was reversed in the β absolute band power, which was higher in the posterior area in the healthy control group, and lower in the critically ill and non-critically ill subgroups. Increased power in slower frequency bands (δ and θ) and decreased power in faster frequency bands (α and β) are seen with reductions in brain metabolism (22). No previous studies have reported anterior-to-posterior gradient changes in electrophysiology in anti-NMDAR encephalitis; however, several FDG-PET/CT-based studies have observed specific anterior-to-posterior metabolic gradient changes in the active phase of NMDA encephalitis, and reported these to correlate with disease severity and renormalize with treatment and recovery (23–25). The observed anterior-to-posterior gradient may largely be driven by posterior hypometabolism rather than anterior hypermetabolism (25). Wegener et al. have identified a predominant pattern of frontotemporal hypermetabolism and parietal hypometabolism (26). However, they found that there were no consistent results regarding FD-PET results and impairment as indicated by the mRS (26), which is consistent with our study relative to the prognostic value of the anterior-to-posterior gradient. Another FDG-PET/CT-based study demonstrated marked posterior hypometabolism in patients with anti–NMDAR encephalitis with severe neurologic disability (mRS 4–5), which was more evident than in those less neurologically disabled (mRS 0–3) (25). In our study, the posterior δ absolute band power in non-critically ill subgroup is lower than the anterior area, and this discrepancy disappeared in the critically ill subgroup, which may indicate that more a significant posterior hypometabolism emerged in the critically ill subgroup and supports the research of Probasco et al.

The main limitation of our study is the small number of patients, which limits the power of the findings. In addition, patients requiring qEEG monitoring due to decreased consciousness or suspected seizures, but were not severe enough to require ICU admission, were enrolled as the non-critically ill subgroup. This might have led to a selection bias; however, it also enabled the analysis of the most challenging group of patients with this disease, in whom prognostic biomarkers are most needed. Additional analysis of temporal and occipital areas, as well as prolonged qEEG monitoring, are needed in the future. Furthermore, while critically ill patients are monitored, some were being administered with anti-epileptic drugs, sedatives, or antipsychotics at the same time. In our study, almost all patients in the ICU was administered at least one intravenous sedative, including midazolam, diazepam, or propofol, to control seizures and involuntary movements in the early course of the disease. Before the start of qEEG monitoring, we requested that these sedatives be suspended and restarted after the monitoring is over. However, these medications may still have an impact on EEG signals. In general, sedatives and antiepileptic medications depress the electrocortical activity and render the EEG background more discontinuous and depressed than expected; therefore, a continuous background may become slightly discontinuous (27). If the EEG background is considered normal there is typically no problem with interpretation. In this study, the aEEG lower margins of all patients were continuous (>5 μV); therefore, sedatives and anti-epileptic medications were unlikely to have had a significant impact on our EEG results.

In conclusion, the qEEG pattern in anti-NMDAR encephalitis can offer better understanding of the pathophysiological mechanisms and prognostic possibilities. A wider parietal aEEG bandwidth was associated with worse long-term outcomes, and may serve as a useful biomarker in anti-NMDAR encephalitis. Further, well-designed studies are needed to confirm this novel finding, and elucidate the underlying mechanism.

## Author contributions

NJ wrote the initial draft of the paper. HG and HR acquisitioned patients' demographic and clinical data from encephalitis database registration. QL guided for analyzing the EEG signals. BP guided for study designation and made critical revision of draft.

### Conflict of interest statement

The authors declare that the research was conducted in the absence of any commercial or financial relationships that could be construed as a potential conflict of interest.
